# CYTOP Fibre Bragg Grating Sensors for Harsh Radiation Environments

**DOI:** 10.3390/s19132853

**Published:** 2019-06-27

**Authors:** Christian Broadway, Damien Kinet, Antreas Theodosiou, Kyriacos Kalli, Andrei Gusarov, Christophe Caucheteur, Patrice Mégret

**Affiliations:** 1 Telecommunications and Electromagnetism Department, University of Mons, Blvd Dolez 31, 7000 Mons, Belgium; 2 Cyprus University of Technology, Limassol 3036, Cyprus; 3 SCK•CEN, Belgian Nuclear Research Centre, 2400 Mol, Belgium

**Keywords:** polymer optical fibres, gamma radiation, fibre Bragg gratings

## Abstract

We present a polymer fibre Bragg grating sensor and its sensitivity to gamma radiation by observing the reflected spectral profile. The Bragg grating is femtosecond inscribed within a perfluorinated CYTOP fibre and the alteration of the Bragg wavelength corresponds to the total radiation dose received. Over a total dose of 41 kGy, the fibre demonstrates a sensitivity of −26.2 pm/kGy and a resolution of 40 Gy. Under active consideration for the instrumentation of nuclear waste repositories, this study gives a better understanding of the effects of gamma radiation upon Bragg gratings in CYTOP fibres.

## 1. Introduction

Numerous practical applications exist for sensors that tolerate and withstand irradiation in domains such as nuclear energy, waste storage and medical treatment. The variety of dose rates and measurands have led to an immense and growing variety of conventional and optical sensors.

Nuclear waste repositories require a range of monitoring technologies for parameters such as buffer integrity, humidity, corrosion, hydrogen levels and temperature. Their sensing needs span the two final phases of operation: stockage and post-closure. The post-closure operational phase is of particular difficulty, with complex power and data transmission needs and solutions. Long-term sensor operation without the possibility of replacement is also a significant factor. As a result, many of the sensing techniques focus on the stockage phase, a period of roughly 50 years when a part of the facility is loaded with radioactive materials and then sealed off when full. Monitoring during this time period is in a standard atmosphere with easy access at any given time, lowering the application complexity. Within the context of MODERN-2020, a project focusing on nuclear waste repositories, analyses indicate that solely gamma radiation is present in these storage cells. A good example of ongoing work includes that of Lesoille et al. [1], who discussed quasi-distributed measurements for dosimetry, hydrogen, and temperature monitoring within a demonstrator of this application context.

Optical fibre sensors have already been demonstrated for dosimetry [2] and temperature sensing [3], among many others. In terms of quasi-distributed sensing, fibre Bragg gratings (FBGs) are commonly used in this context, as they can deliver single digit centimetre precision dependant upon the equipment and parameters employed. These intrinsic fibre structures have been inscribed in silica fibre for over 30 years [4] and are favoured for leveraging the intrinsic properties of the fibre in question. Bragg gratings have been implemented for applications including structural health monitoring [5,6], hydrogen detection [7] and ultrasonics [8], rendering them applicable and of interest for the domain of nuclear waste repository monitoring.

The effect of gamma radiation on silica fibres is well documented [9,10], where the degradation of colour centres within the fibre causes a marked increase in optical attenuation [11]. Polymer optical fibres (POF) are an alternative that have only recently been under consideration for these applications. The bulk of these results are for X-ray dosimetry [12,13], although other contributions have gone some distance towards defining radiation induced attenuation (RIA) [14,15] and bulk material degradation [16]. While the RIA of CYTOP fibres has been considered up to certain gamma radiation doses, it is important to understand and consider the practical impact of gamma radiation on the FBG itself.

In recent years, POF has become more interesting as a widening variety of polymer types leverage a veritable catalogue of different fundamental properties. Polymers have larger elastic limits than silica fibres and are generally more strain and temperature sensitive. PMMA is highly humidity sensitive [17], TOPAS is effectively humidity insensitive [18], polycarbonate has a higher heat tolerance with those same advantages [19] and CYTOP is a low loss polymer in the 1550 nm wavelength region of the optical spectrum that allows the use of tens of metres of fibre [20]. Many papers based on these polymers have already demonstrated that silica fibres are outmatched in terms of fundamental properties and a growing interest in polymers has been observed as a consequence. Nonetheless, individual polymers present weaknesses that are often associated with commercial readiness or, alternately, present sensitivity to variables that are desirable for few applications such as humidity.

To date, PMMA and CYTOP have both been considered in one form or another with respect to radiation environments [12,13,15]. In the context of distributed sensing for monitoring within structures such as nuclear waste repositories, CYTOP presents the advantage of longer operational distances than other polymer fibres and could even be considered at the present time as the only practical polymer fibre for distributed sensing in 1550 nm region [21], this band being of interest for the wide range of compatible equipment and the often lower prices for components such as optical circulators. Studies by the group of Stajanca and Krebber have discussed the impact of radiation on CYTOP fibres [15,22], examining the attenuation of pristine fibres across the optical spectrum. However, further knowledge is necessary before using CYTOP FBGs for monitoring in irradiated environments, starting with the evaluation of FBGs and connectorisation technologies under gamma radiation.

We present the effects of gamma radiation on a CYTOP FBG with active temperature and passive humidity control, showing that CYTOP can function in radiation environments and be a viable platform for distributed sensing. We analyse the physical and optical impact of gamma radiation on the FBG and set out future work that will render monitoring in radiation environments more effective using CYTOP polymer fibres.

## 2. Materials and Methods

### 2.1. Fibre Preparation

CYTOP is a commercially certified perfluorinated graded-index polymer fibre branded as GIGAPOF and sold by Chromis Fiberoptic. The fibre used for this work has a core diameter of 120 μm, 20 μm cladding and is jacketed in polycarbonate for a total fibre diameter of 490 μm, said jacket being for enhanced mechanical stability. GIGAPOF is the most common brand of CYTOP to be reported on scientifically and presents a more cost effective baseline from which to evaluate the field of perfluorinated fibres in general. An FBG was inscribed at Cyprus University of Technology using the plane by plane femtosecond inscription technique [20]. To obtain a quasi-single-mode Bragg grating, the maximum grating length was restricted to 1 mm, the grating length was chosen to be 1 mm to maximise grating peak power.

**Figure 1:**
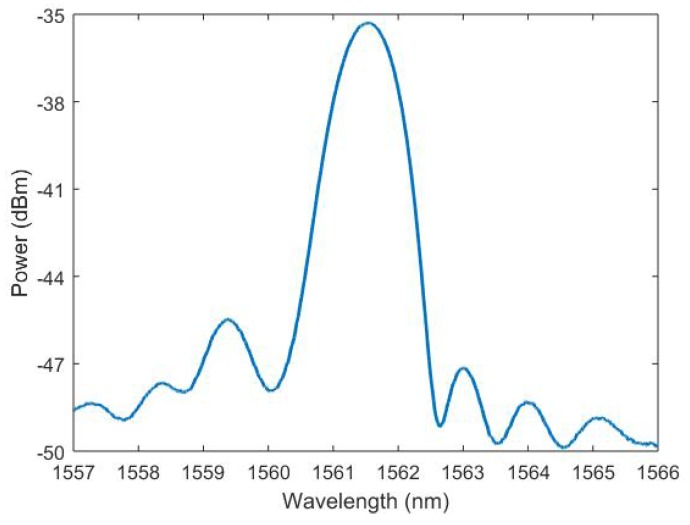
Spectral profile of the CYTOP FBG within the test container.

Two options were presented for monitoring the radiation induced changes throughout the course of the experiment. The fibre could be examined pre- and post-irradiation using butt coupling techniques, or the fibre could be spliced using UV gel to a silica fibre and tracked on-line throughout the experiment. While the first option would remove the on-line possibility of data measurements, the second option would introduce the splicing gel element, a potentially sensitive coupling joint within the reach of gamma radiation. While the addition of a connection material brings additional elements into the analysis, on-line measurements were considered vital to truly understand the FBG response and important to consider in their own right for radiation environments. The CYTOP fibre was connectorised to silica SMF-28 fibre using the UV gel curing technique. Our variation of this technique typically delivers strong adhesion with minimal variance and low optical losses [23]. The resulting splice was separated from the FBG by over 20 cm of fibre to allow limited bending without affecting coupling conditions or pre-straining the FBG. The reflected spectral profile of this FBG in Figure 1 was obtained using a Micron optics interrogator (NI PXIe-4844).

### 2.2. Gamma Irradiator Preparation

The application context anticipates a pure gamma environment with a steady discharge of gamma radiation over a 50-year period. The irradiation at SCK•CEN was performed in the underwater irradiation facility RITA (Radio Isotope Test Arrangement). RITA is located at the bottom of a pool with circa 7 m of water that serves as a radiation shield. It consists of a frame with Co-60 source holders, the guiding device which transports the irradiation container between a position above the water surface and the underwater irradiation position, and instrumentation panels. The cylindrical water-tight irradiation container has a diameter of 380 mm and a 600 mm height. The container cover permits the attachment of flexible hoses to enable on-line measurement capabilities by permitting standard silica optical fibres to connect interrogator and fibre.

**Figure 2:**
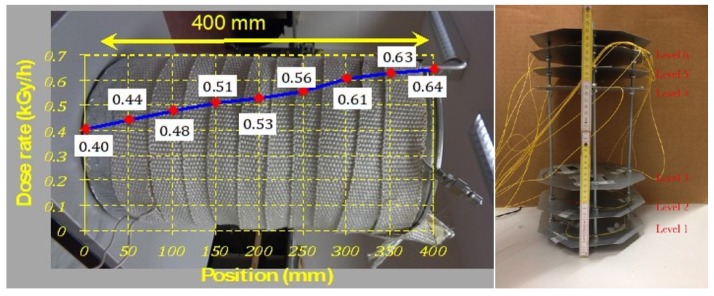
Dose distribution of gamma radiation over the container kGy/h (**Left**). Custom mount, with the CYTOP fibre placed on the 6th level (**Right**).

For the test, eight cylindrical Co-60 sources were symmetrically arranged around the irradiation container, creating an omnidirectional radiation field. The height of the sources is 13 cm and the vertical dose-rate profile presenting a hat-shape form with a 15 cm flat region where dose-rate variations are within 10%. On horizontal planes, the dose-rate variations are within 10% for a 30 cm diameter circle. The dose-rate distribution in the irradiation container was mapped less than three weeks prior to the irradiation using Harwell Amber 3042 dosimeters, traceable to the UK standard for absorbed doses at the UK National Physical Laboratory. The standard accuracy of this method, which includes batch variations and the calibration uncertainty, is ±6% and the results are shown in Figure 2.

The standard RITA instrumentation includes temperature measurement and atmosphere control. The present irradiation was performed in air. The temperature was automatically regulated using feedback from a thermocouple inside the oven. The initial temperature was first set slightly above the target for irradiation and then decreased during a stabilisation period prior beginning the irradiation to the irradiation temperature of 30.1 °C ± 0.1 °C. This value allows obtaining stable conditions similar to room temperature while taking into account the effect of radiation heating.

We implemented a mounting system to provide precise height calibration in order to deliver doses with maximum precision, as shown in Figure 2 (Right). To optimise irradiator time, multiple experiments were placed at different heights on the mount; we omit references to these fibres as they are out of the scope of this project. To test fibres accurately, they were first secured at designated heights on our mount to obtain specific irradiation dose rates, with a reflected spectral profile taken once secured. The mount was then inserted into the test container (as shown in Figure 2 (Left)) along with the thermocouple. The test container was then placed into a larger, hermetically sealed container and immersed into the pool to begin a stabilisation period prior to irradiation.

### 2.3. Fibre Installation

To track temperature effects, perceive source power fluctuations and correctly attribute localised disturbances, we placed a silica reference fibre with an inscribed FBG in an external oven in the RITA control room and connected it to our interrogator, outside of the irradiation zone. The oven was stabilised to the same temperature as the test container for the duration of the experiment. This permitted us to verify temperature stabilisation times and values, allowing us to state with a degree of certainty whether temperature changes play any role in a given change in FBG performance.

The connectorised CYTOP fibre was installed on level 6 of our mount to obtain a dose rate of 635 Gy/h. The fibre splice was secured using a cardboard support during connectorisation to avoid deformation that could change coupling conditions, ensuring that the fibre joint is straight without applying additional force. The fibre was secured using tape to the metal level platform, also without applying strain.

Prior to the start of irradiation, temperature control was initiated and the FBG allowed to stabilise for 1.5 h. At the conclusion of the experiment, a rest period without irradiation was accorded to observe spectral recovery or the lack thereof. We used a Micron Optics 4 channel optical interrogator (NI PXIe-4844) with a 1 pm resolution to obtain these profiles prior to and during the experiment. We saved the results automatically at 250-s intervals throughout the test, from the start of the stabilisation period to the end of the rest period. We subjected the fibre to two irradiation runs, the first of 41 kGy and the second an additional 79 kGy for a total dose of 120 kGy.

## 3. Results

Our objective in preparing this study was to determine the effect of gamma radiation on CYTOP fibre with an inscribed FBG. Given our choices of experimental plan, this also included the effects of UV gel for coupling under the same conditions. We controlled humidity passively and actively control the temperature through a feedback loop, ensuring no additional strain would be added during the experiment through careful installation practices. To achieve this objective, we established the effects of gamma radiation by examining physical and spectral changes on our installed and irradiated fibre. This is then discussed in relation to our application context of nuclear waste repository monitoring, where this test formed the basis for whether or not CYTOP FBGs can survive in such an environment.

### 3.1. Physical Changes

**Figure 3:**
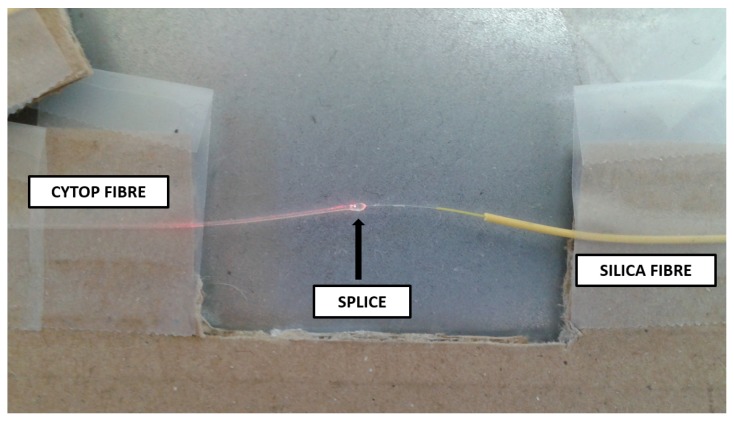
Physical changes in CYTOP fibre after 120 kGy gamma radiation, illuminated using a 632 nm light source.

We examined the fibre after the total dose of 120 kGy. While the ceramic fibre connectors darkened in colour, the CYTOP fibre was visibly unchanged and appeared as it did prior to the tests. Likewise, the UV gel connection appeared the same. However, significant deformation of the CYTOP fibre occurred in the splice region where the fibre was previously held straight. This is shown in Figure 3.

We observed an extremely significant deformation, despite the support being intact. We could immediately eliminate the silica fibre as part of the cause as the effects on silica fibre were well established. Having examined PMMA polymer fibres connected using the same UV curing method and presented in the same irradiation campaign, we could discard the UV gel as a cause of the deformation. We theorise that the CYTOP fibre and/or the polycarbonate jacket responded adversely to gamma radiation by physically deforming, but are unable to comment further as the literature does not yet contain a definitive answer on this subject. This subject merits further investigation. Using a 632 nm inspection laser, we could see apparent losses in the region of the splice that should cause increased attenuation. This phenomena had not previously been observed as the scenario of exposing a UV gel splice to gamma radiation had not been attempted to date. These results are interesting for the practical implementation of a CYTOP FBG.

### 3.2. Spectral Changes

**Figure 4:**
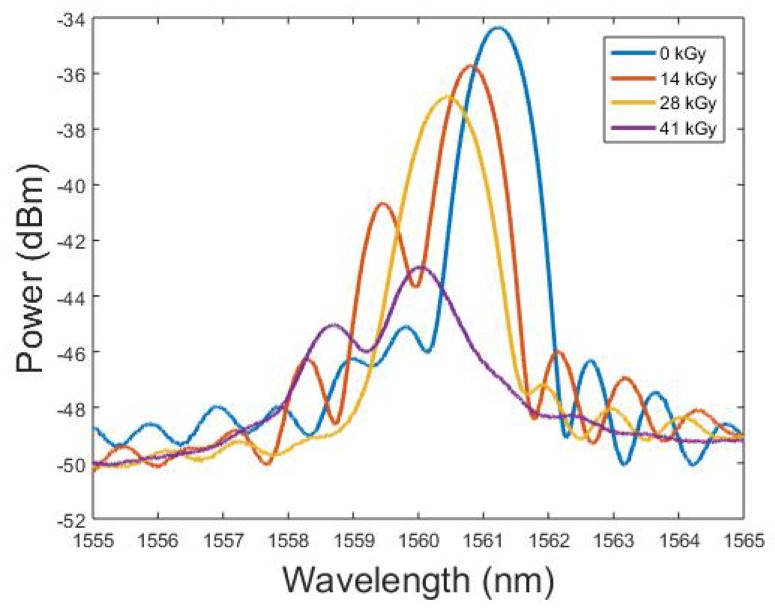
Spectral profile changes under gamma radiation.

We examined the peak amplitude and wavelength changes of the FBG over the first 41 kGy. The shape of the FBG profile in general was first considered by taking profile images at regular intervals and comparing them in Figure 4.

The FBG profile underwent power and shape changes with the two sub-peaks appearing to increase in power and merge by 14 kGy, only to merge in turn with the primary peak at 28 kGy. By the time the fibre received a total dose of 41 kGy, we observed a significant loss of profile shape and peak power, along with the return of a sub-peak of increasing power. When considering how to analyse and accurately determine the changes in Bragg wavelength, any identification based on full-width-half-maximum (FWHM) or similar techniques would be ineffective as for higher doses, the sub-peak was within 3 dB of the primary peak. Based on the profile changes, taking the Bragg Wavelength value directly was preferable. The amplitude of the spectral profile was also of interest, especially in the context of the physical observations previously mentioned.

**Figure 5:**
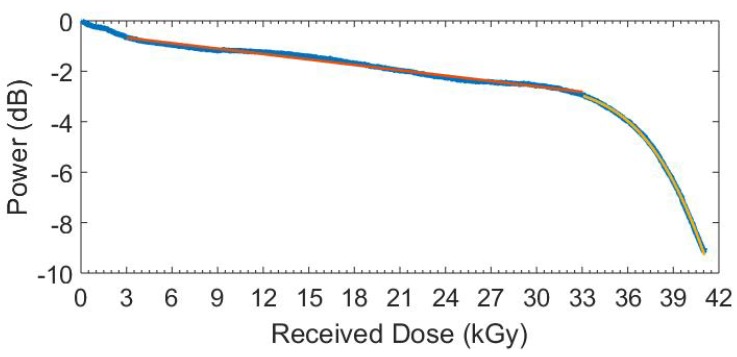
Amplitude degradation of the Bragg wavelength in CYTOP (blue), linear fit for first section (red) and cubic fit for the second section (yellow).

We analysed the amplitude response of the FBG, as shown in Figure 5, where a sudden and rapid amplitude decrease near the end of the irradiation period heralded imminent coupling failure. Complete coupling failure occurred shortly after, during the stabilisation period of the final 79 kGy and prior to further irradiation. As a result, we analysed only the first 41 kGy, with the FBG no longer visible at the interrogator after this point.

We observed a steep negative gradient within the first 3 kGy, but, having verified the results from our silica reference FBG, there was a continuing temperature stabilisation effect within this period that may affect the amplitude results. As a precaution, we removed the first 3 kGy from our consideration. The amplitude changes can be summarised as a small negative decline of 3 dB from 3 to 33 kGy and a steep negative decline from 33 to 41 kGy where the amplitude decreased by over 6 dB. These discrete sections are shown with linear and cubic fits, respectively. The first fit has an $R^{2}$ of 0.988 and the second an $R^{2}$ of 0.999, with the respective equations of −0.072x−0.46 and $-0.006x^{3}+0.561x^{2}-17.948x+190$. The gradient for the first zone is −0.07 dB/kGy and −1.87 dB/kGy for the second zone at 40 kGy. As there was no steady decline and having observed physical deformations that would normally increase optical losses, we investigated the possibility of a connection between the two phenomena. However, while the amplitude losses are valuable and of interest, we highlight that the wavelength changes were unaffected by amplitude changes until the attenuation becomes high enough to mask the FBG profile.

**Figure 6:**
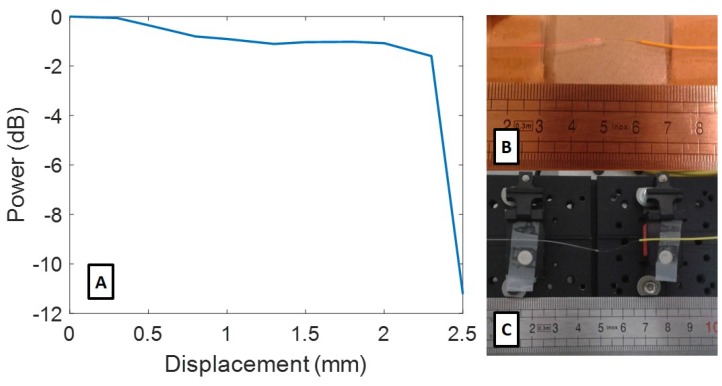
Amplitude changes for the deformation on a connection joint in the CYTOP fibre (**A**); state of a coupling joint after 120 kGy of radiation (**B**); and state of a pristine coupling joint under mechanical deformation (**C**).

Considering the possibility of the observed physical changes in the splice region influencing the amplitude response, we prepared a pristine CYTOP FBG and subjected the splice region of the fibre to a mechanical compression in steps until the irradiated deformation was replicated. We tracked the amplitude relative to the noise level, as shown in Figure 6A, with the duplicated deformation shown in Figure 6C.

There was a notable similarity between the amplitude response of the irradiated fibre and the pristine fibre with an induced coupling joint deformation. While it is unclear exactly what deformation was attained before and after 41 kGy, the similarity provides a good explanation and correlation between the observed physical deformation and the amplitude attenuation. It is important to note that these amplitude changes had no direct impact on the FBG wavelength, thus did not compromise the measurement technique or its precision. However, the amplitude changes may render the FBG effectively invisible, as they did after the 41 kGy mark in this irradiation campaign.

**Figure 7:**
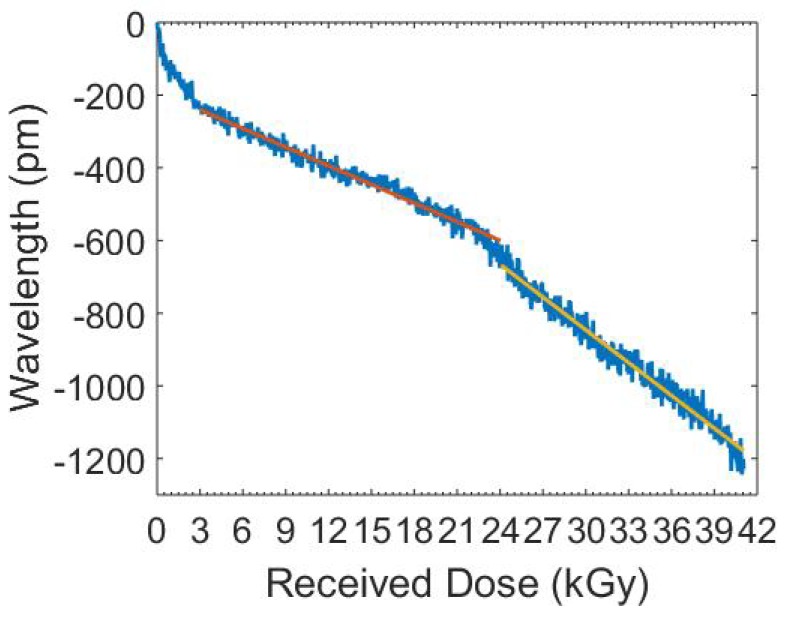
CYTOP FBG Bragg wavelength shift due to Gamma radiation (blue), linear fit for first section (red), and linear fit for second section (yellow).

We considered changes in the Bragg wavelength shown in Figure 7, where the Bragg wavelength underwent a significant negative shift. Establishing a sensitivity based on the start and end points of the data, we obtained −29.9 pm/kGy. However, there was a significant and visual impact within the first 3 kGy dose, which we attributed to temperature stabilisation. We discarded this data segment as a consequence when considering sensitivity to gamma radiation.

Prior to evaluating the radiation response with respect to the modified dose parameters, we could also extract useful information to contribute towards the ongoing discussion on the temperature sensitivity of CYTOP fibres. CYTOP has been attributed a positive thermo-optic coefficient in some papers [24], but the un-stripped fibre is susceptible to the reactions of the significant polycarbonate jacket, a material with a negative thermo-optic coefficient [25]. We observed a sharper negative gradient in our Bragg wavelength shift while a known positive temperature increase occurred and then remained stable. Furthermore, our experiment had no plausible source of strain or humidity alteration. Our fibre appeared to display the characteristics of a fibre with a negative thermo-optic coefficient and brought CYTOP into harmony with all other polymer fibres in this respect. Further research in this domain would be profitable, but is out of the scope of this study.

Considering in more detail the sensitivity of our fibre to gamma radiation and starting from a dose of 3 kGy, we obtained an overall linear sensitivity of −26.2 pm/kGy. Careful examination indicated two phase shifts, the first from 3 to 24 kGy and the second from 24 to 41 kGy. The first region could be described using a linear fit with an $R^{2}$ of 0.967 and an equation of −17.1x−189.2, with a sensitivity of −17.1 pm/kGy. The second region was described using a linear fit with an $R^{2}$ of 0.966 and an equation of −30x+56, yielding a larger sensitivity of −30 pm/kGy. This distinction suggests that gamma radiation affected the fibre in at least two distinct ways and, at higher doses, this led to a more rapid wavelength shift. This analysis would be profitably employed serving as the basis for future studies into CYTOP fibres.

Given the 1 pm resolution of our interrogator, our FBG has a resolution to gamma radiation slightly below 40 Gy. In a practical environment, variables such as temperature, strain and humidity also dictate the resolution of a detector. Temporarily ignoring wavelength shift direction, the total wavelength shift of 1 nm seen in this test is equivalent to a 70% increase in relative humidity, the application of 700 μϵ or a 55 °C increase in temperature [26]. Gamma radiation therefore has a more significant impact on CYTOP FBGs than these variables in ambient environments, recommending them for consideration as dosimeters.

## 4. Discussion

Our results present the first on-line radiation study of polymer fibres using FBGs. To provide context and discuss our results relative to current research, we consider the work of Stajanca et al., whose research covers CYTOP fibres across a broader telecommunications spectrum without intrinsic sensors. Stajanca et al. [15] treated the issue of gamma radiation for the smaller CYTOP 50 μm core GIGAPOF, including its effect on thermal stability and humidity sensitivity. The reported data show that the RIA at 1550 nm attains 12 dB/m after 100 kGy of gamma radiation, with humidity-based attenuation increasing. While no direct study on the origins of RIA within CYTOP exists, Stajanca et al. explored some aspects of the mechanisms that may be behind the observed RIA and proposed CYTOP for radiation sensing. It is difficult to compare RIA with the observed amplitude changes in reflection of an FBG where attenuation values are not absolute, as opposed to measurements in transmission. The increased humidity related attenuation is also difficult to apply in this context, as attenuation only affects the FBG when it becomes severe enough to allow the noise level of the system to mask the FBG profile. Considering the values reported by Stajanca, the values are not severe enough to cause concern, especially given the amplitude and wavelength results presented in this manuscript. Our study makes observations for the first time regarding femtosecond inscribed FBG interactions in CYTOP fibre under gamma radiation and, while the profitable studies of Stajanca are also in CYTOP fibre, it is not yet possible to draw specific conclusions about the physical mechanisms contributing to the negative wavelength shift that we observe. New campaigns based on these results and with the objective of identifying the physical mechanisms would be necessary to make definitive conclusions.

## 5. Conclusions

We have presented a femtosecond inscribed quasi-single-mode FBG within a CYTOP fibre that was connectorised and exposed to gamma radiation in excess of 40 kGy under an online testing regime. Our work shows that an FBG point sensor can function in this region, detailing the physical and optical changes. We calculated an overall sensitivity of −26 pm/kGy to gamma radiation and a resolution of less than 40 Gy with the mentioned interrogator and permit consideration to be given to radiation compensation. A challenge was identified with fibre connectorisation, where the effects of radiation cause significant coupling deformation resulting in total coupling loss. We demonstrated that CYTOP FBGs may be advantageous for nuclear waste repository sensing along with other radiation environments, both as a gamma radiation sensor as well as other measurands such as strain. New campaigns could further this work by exploring the physical mechanisms of the observed changes and resolve the current challenge with connection strength. Sufficient mitigation would allow any results to be limited uniquely by the FBG and fibre properties, increasing the maximum survivable dose and the quality of the sensor response.
